# DNA Repair and Therapeutic Strategies in Cancer Stem Cells

**DOI:** 10.3390/cancers15061897

**Published:** 2023-03-22

**Authors:** Matthew S. Gillespie, Ciara M. Ward, Clare C. Davies

**Affiliations:** 1 Institute of Cancer and Genomic Sciences, University of Birmingham, Birmingham B15 2TT, UK; m.gillespie@soton.ac.uk (M.S.G.);; 2 School of Cancer Sciences, University of Southampton, Southampton SO16 6YD, UK

**Keywords:** cancer stem cells, breast cancer stem cells, tumour-initiating cells, DNA damage response, DNA repair, chemoresistance, radioresistance, homologous recombination, non-homologous end-joining, splicing

## Abstract

**Simple Summary:**

A major theory of cancer development is that cancer originates from a specialised type of tumour cell called the cancer stem cell (CSC). Although CSCs comprise a relative subpopulation to the overall heterogeneous tumour mass, they are responsible for cancer establishment, progression, metastasis, and relapse. The eradication of CSCs is therefore vital for long-term patient remission and survival; however, achieving this is challenging as these cells are highly drug resistant compared to the bulk of cancer cells. CSC drug resistance is multifaceted, occurring through multiple extrinsic and intrinsic mechanisms, including an improved ability to repair chemo/radiotherapy-induced DNA lesions. This review summarises the evidence supporting the notion that CSCs display enhanced DNA repair efficiency relative to the bulk tumour population, the possible mechanisms by which this occurs, and discusses strategies of targeting the DNA damage response within CSCs to improve the efficacy of cancer treatment.

**Abstract:**

First-line cancer treatments successfully eradicate the differentiated tumour mass but are comparatively ineffective against cancer stem cells (CSCs), a self-renewing subpopulation thought to be responsible for tumour initiation, metastasis, heterogeneity, and recurrence. CSCs are thus presented as the principal target for elimination during cancer treatment. However, CSCs are challenging to drug target because of numerous intrinsic and extrinsic mechanisms of drug resistance. One such mechanism that remains relatively understudied is the DNA damage response (DDR). CSCs are presumed to possess properties that enable enhanced DNA repair efficiency relative to their highly proliferative bulk progeny, facilitating improved repair of double-strand breaks induced by radiotherapy and most chemotherapeutics. This can occur through multiple mechanisms, including increased expression and splicing fidelity of DNA repair genes, robust activation of cell cycle checkpoints, and elevated homologous recombination-mediated DNA repair. Herein, we summarise the current knowledge concerning improved genome integrity in non-transformed stem cells and CSCs, discuss therapeutic opportunities within the DDR for re-sensitising CSCs to genotoxic stressors, and consider the challenges posed regarding unbiased identification of novel DDR-directed strategies in CSCs. A better understanding of the DDR mediating chemo/radioresistance mechanisms in CSCs could lead to novel therapeutic approaches, thereby enhancing treatment efficacy in cancer patients.

## 1. Introduction

Cancer is one of the leading causes of mortality worldwide. Although emerging treatments aim to target genetic/epigenetic vulnerabilities enabling patient stratification, the majority of research—and hence, therapies—has not considered the cancer stem cell (CSC) population. This is an important cellular subpopulation because CSCs distinctly influence a patient’s response to treatment. The concept of CSCs and their distinct tumourigenic properties was first introduced in pioneering studies investigating acute myeloid leukaemia (AML) engraftment in immunocompromised mice [1,2]. The authors demonstrated that not all tumour cells were cancer-initiating and that a rare subpopulation (0.1–1% of tumour cells) was responsible for tumour formation and disease progression [1,2]. Importantly, these “tumour-initiating” cells were characterised by the CD34^+^CD38^−^ surface marker expression profile, which also defines normal haematopoietic stem cells (HSCs) and thus subsequently termed the CSC population [2]. The concept of a tumour-initiating/CSC population has now been proposed for many solid cancers including breast, prostate, brain, colorectal, lung, and pancreas [3,4,5,6,7,8].

Currently, the CSC model of cancer development postulates that tumours are hierarchically organised based on differentiation capacity and maintained by a fraction of cells responsible for cancer formation and tumour heterogeneity [9,10]. In contrast, the clonal evolution theory states that all cancer cells have the potential to be tumour-initiating and that those mutations offering a survival advantage ultimately lead to clonal outgrowth and tumour heterogeneity [11]. CSCs have characteristics in common with normal adult stem cells including self-renewal, unlimited proliferative capacities, and an ability to generate cell progeny capable of differentiating and proliferating rapidly. As such, it is believed that CSCs are generated via the transformation of somatic stem/progenitor cells. Indeed, adult stem/progenitor cells gradually accumulate mutations over time with targeted germline oncogenic transformation of the normal stem or progenitor cell promoting tumorigenesis in mice [12,13,14]. This concept is also supported through in vivo lineage tracing of the transformed stem cell population and barcoding of patient-derived glioblastoma cells and xenograft transplantation [12,13,15]. However, the precise source of CSCs appears to be more complex and multifactorial because CSCs from carcinomas characteristically display an epithelial-to-mesenchymal (EMT) gene signature and phenotype. It is now known that CSCs can also arise from the dedifferentiation of a bulk tumour cell to one exhibiting stem cell-like characteristics via EMT induction, particularly after tissue damage [16,17,18]. This plasticity of CSCs has significant implications for cancer therapy and infers the need for combination approaches that target both CSCs and the bulk tumour population.

A final consideration is the identification and isolation of CSCs. While markers are well-defined for leukaemic CSCs, identifying universal CSC markers for specific carcinomas has been challenging due to the inherent presence of tumour subtypes and intratumoral heterogeneity [3,13,19,20,21,22,23,24]. For example, the most common methods to isolate breast CSCs (BCSCs) are through either EpCAM/CD24/CD44 cell surface marker expression or ALDH1 (aldehyde dehydrogenase 1) activity [3,24] (Table 1). Using these markers, it was found that BCSCs derived from basal-like breast cancers were more mesenchymal-like expressing CD24^low/−^CD44^+^, whereas luminal ER^+^ breast cancer subtypes contained an enriched population of ALDH1^+^ BCSCs and a diminished fraction of CD24^low/−^CD44^+^ BCSCs [25]. Additionally, within the same tumour, these mesenchymal-like CD24^low/−^CD44^+^ BCSCs were shown to be located at the tumour front compared to the less invasive epithelial-like ALDH1^+^ BCSCs which predominately localised to the tumour core [26]. More recent analysis using single-cell RNA sequencing (scRNA-Seq) has also identified that the heterogeneity within BCSCs is organised hierarchically [27]. The use of pre-defined CSC biomarker criteria is therefore limited with respect to fully encompassing the CSC phenotype within a given tumour. Viable alternative approaches to isolate or enrich CSCs utilise functional parameters and include anchorage-independent cultures, hypoxia enrichment and the uptake of the lipophilic PKH26 red-fluorescent dye. Notably, these enriched CSC populations are known to express a stemness gene signature and exhibit increased in vivo tumour-initiating capacities [28,29,30]. Furthermore, CSCs are operationally defined based on their long-term self-renewal potential in xenograft studies. Indeed, the limiting dilution analysis, which identifies the number of tumour-initiating cells, has long been considered the gold standard in vivo assay for CSC characterisation; however, it also has important caveats that require consideration.

According to the CSC model, tumour-initiating cells possessing stem-cell like behaviour are hypothesised to be more chemoresistant than the bulk cancer cell population, and that inability to eradicate CSCs increases the probability of tumour relapse and metastatic growth. While there are multiple mechanisms at play that contribute to CSC drug resistance (as discussed in Section 2), one that has yet to be fully explored in depth is how CSCs exploit genome stability mechanisms for their survival. Indeed, it has been suggested that CSCs are resistant to genotoxic treatments because they possess a more robust DNA damage response (DDR) compared to their bulk progeny resulting in improved repair of cytotoxic DNA lesions, contributing to increased cell survival. Despite this, the repair pathways that CSCs are dependent on and the mechanisms by which elevated DNA repair activity is achieved is still unresolved. Hence, therapeutic targeting via the DDR in CSCs remains relatively unexplored. Given the recent developments of highly specific DDR inhibitors coupled to advances in targeting DNA repair pathways via synthetic lethality approaches in bulk tumour cells [31], CSCs exhibiting dependencies on specific DDR pathways for countering chemo/radiotherapy toxicities may present a potential “hidden gem” with respect to CSC-directed therapies. In this review, we summarise the current knowledge concerning mechanisms of enhanced genome stability in CSCs and discuss opportunities that exploit knowledge of the DDR in CSCs for therapeutic intervention.

## 2. Therapy Resistance in CSCs

**Figure 1:**
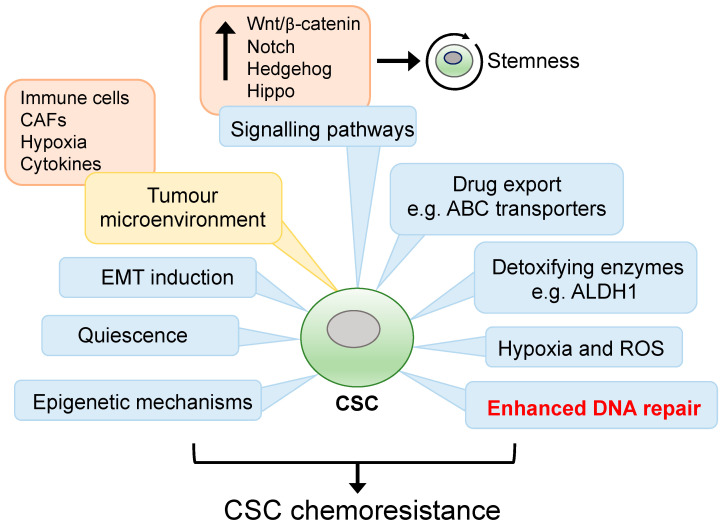
Mechanisms of drug resistance in CSCs. CSCs are endowed with multiple innate and adaptive cell responses which enable them to survive stressful microenvironmental changes and cancer treatment. Enhanced DNA repair efficiency in CSCs is thought to be a major contributing factor in counteracting treatment-induced DNA damage. Cell-autonomous mechanisms are in blue boxes and extrinsic influences are in yellow boxes.

CSC drug resistance can be attributed to intrinsic mechanisms resulting from inherent or acquired genetic/epigenetic alterations, and extrinsic influences mediated by the microenvironment and the stem cell niche [32]. Intrinsic CSC drug resistance mechanism includes increased expression of detoxifying enzymes and drug transporters, epigenetic remodelling promoting stemness pathways, metabolic reprogramming, EMT induction, enhanced DNA repair and quiescence [32,33,34,35] (Figure 1). These cellular processes are influenced by external factors involving autocrine and paracrine interactions between CSCs and various cell types within the local microenvironment. For instance, growth factor and cytokine secretion from immune and endothelial cells, adipocytes, and cancer-associated fibroblasts (CAFs) within a hypoxic tumour microenvironment are known to facilitate a niche supportive of CSC self-renewal, plasticity, and chemoresistance [33,36]. The CSC niche is also thought to promote immunosuppressive properties as the degree of tumour stemness has been linked to reduced CD8^+^ T cell infiltrates and increased immunosuppressive tumour-associated macrophages (TAMs) [37,38,39]. Moreover, EMT induction can result in expression of the immune checkpoint ligand PD-L1. Indeed, high expression of PD-L1 has been observed in breast, lung and head and neck CSCs, which contributed to enhanced antitumoral immune evasion [40,41,42]. Additionally, CSCs are known to express low levels of the major histocompatibility complex class I, thereby reducing antigen presentation, and preventing cytotoxic T cell recognition while also upregulating the anti-phagocytotic cell surface protein CD47 [43].

A major cell-autonomous property of CSCs that contributes to their increased drug resistance is a reduced cell cycle rate compared to bulk tumour cells. Therefore, anticancer agents that rely on high mitotic activities for cytotoxicity are less effective in CSCs as the reduced cell cycle kinetics enables an increased capacity for drug metabolism/detoxification and enhanced repair of drug-induced DNA lesions. For example, elevated ALDH1 activity in slow-cycling HSCs/progenitor cells increased the conversion rate of alkylating chemotherapeutic agents such as cyclophosphamide into non-toxic carboxylic metabolites [44]. In addition, overexpression of ATP-binding cassette (ABC) transport protein family members, such as ABCG2, enables the rapid expulsion of chemotherapeutic drugs and are highly expressed both in normal mammary stem cells and BCSCs [23,45]. Unfortunately, clinical ABC transporter inhibitor therapy in cancer has proven unsuccessful due to high patient toxicities [46]. Hypoxia is another well-established environmental mediator of drug resistance as isolation of hypoxic breast cancer cells from xenograft models exhibited increased tumorigenicity, quiescence and displayed increased BCSC marker expression compared to non-hypoxic tumour cells. These hypoxic BCSCs showed elevated PI3K/AKT signalling and drug targeting AKT promoted differentiation [47].

Besides intrinsic drug resistance, CSCs are highly plastic and may be generated from non-CSCs when under therapeutic pressure or an altered microenvironment. For example, chemotherapy-induced senescence reprograms non-stem bulk leukaemic cells into CSCs, whilst hypoxia enriches for a CD133^+^ glioma stem cell (GSC) population that displays a more aggressive in vivo growth phenotype [48]. Chemotherapies also promote tissue damage leading to TAM infiltration and secretion of cytokines such as TGFβ, TNFα, CCL20, and IL6, which drive EMT [33,49,50]. For example, in some breast cancer subtypes the *ZEB1* promoter is held in a bivalent state in non-CSCs enabling rapid response to TGFβ, EMT induction, and the conversion of non-CSCs to CSCs [51]. Finally, etoposide, cisplatin, doxorubicin, and gemcitabine induce endoplasmic reticulum stress that induces an EMT phenotype and cell migration through upregulated expression of the transcription factors *SNAIL* and *ZEB1* [52]. Phenotypic plasticity is thus a potent tool that can ensure drug resistance by effectively replenishing the CSC pool. 

Remarkably, chemotherapy can also induce CSC proliferation. Indeed, CD24^low/−^CD44^+^ BCSCs were enriched almost three-fold after chemotherapy regardless of molecular subtype, with surviving BCSCs displaying enhanced self-renewal and xenograft growth compared to cells derived from the untreated tumour [53]. A similar phenotype was observed in relapsed lung cancer patients whereby cisplatin-mediated activation of Notch signalling enrich for CD133^+^ lung CSCs. Hence, treating lung xenograft models with DAPT, a γ-secretase inhibitor that blocks Notch signalling, suppressed the CD133^+^ CSC phenotype [54]. Likewise, the PAX6-GLI1-SOX2 pathway promoted lung CSC expansion after a combination of pemetrexed and cisplatin treatment [55]. Moreover, PARP inhibitors can also lead to CSC expansion, as treating ovarian cancer cell lines with olaparib or rucaparib resulted in an increase in the CD133^+^CD117^+^ CSC population that was allied with elevated ALDH1 activity and increased in vitro sphere-forming capabilities. Although administration of olaparib into xenograft-bearing mice slowed tumour growth, the number of ovarian CD133^+^CD117^+^ CSCs was increased. Interestingly, PARP inhibitor-induced enrichment of ovarian CSCs was independent of *BRCA1* mutational status [56]. Importantly, evidence of CSC expansion in immunocompetent mice has also been observed. For example, administration of temozolomide in a glioblastoma mouse model which expresses the DTK-IRES-GFP transgene driven by the *Nestin* neural stem cell-specific promoter, enabling the demarcation of GSCs, resulted in eradication of the bulk population but expansion of the quiescent GSC subpopulation. As such, depleting the GSC compartment through ganciclovir-mediated lineage ablation was required to effectively delayed the onset of tumour regrowth in mice undergoing chemotherapy, implying that a CSC population is required for tumour regrowth [57].

Although various well-described mechanisms and mediators contribute to CSC drug resistance, the cytotoxicity of radiotherapy and most chemotherapeutic drugs is dependent on the induction of double-strand breaks (DSBs) [58]. Hence, these cells are presumed to possess increased DNA repairability compared to the bulk tumour population which essentially counteracts the cytotoxicity of DNA-damaging antineoplastic agents. Targeting components of the DDR within CSCs therefore presents an attractive strategy to drug re-sensitise CSCs, and as such is the focal point of this review.

## 3. The DNA Damage Response (DDR)

**Figure 2:**
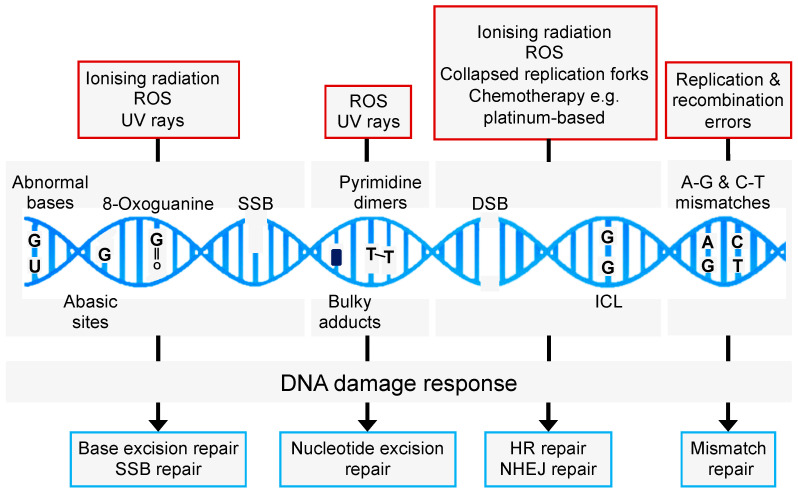
Common causes of DNA damage and repair pathways. Endogenous and exogenous DNA damaging agents promote a range of DNA lesions that are repaired by distinct and overlapping repair pathways. HR: homologous recombination; NHEJ: non-homologous end-joining; SSB: single-strand break; BER: base excision repair; ICL: interstrand crosslink.

Maintaining genome integrity and stability after exposure to endogenous and exogenous DNA damaging agents is essential for cell survival. A failure to trigger an effective DDR leads to mutagenic events and loss of genetic material which predisposes individuals to cancer and neurological diseases. To counteract this, cells have evolved multiple DDR pathways that enable sensing, signalling, and repair of specific DNA lesions (Figure 2). These include mismatch repair (MMR), base excision repair (BER), single-strand break (SSB) repair, nucleotide excision repair (NER), translesion synthesis (TLS), the Fanconi Anaemia (FA) pathway, and DSB repair through non-homologous end-joining (NHEJ) and homologous recombination (HR). 

In comparison with normal cells, cancer cells exhibit an elevated level of DNA damage due to oncogene-induced replication stress (RS) leading to hyperproliferation as well as oncogenic mutations in crucial players of the DDR (e.g., *ATM*, *ATR*, *BRCA1*, *BRCA2*, and *TP53*). Cancer cells therefore rely on an effective DDR for their survival which may manifest into mechanisms of drug resistance. For instance, cisplatin induces the expression of the TLS polymerase *REV3* in lung cancer cell lines leading to cisplatin resistance [59], while cisplatin-resistant lung cancer cells show significant upregulation of the DSB repair genes *RAD51*, *RAD52,* and *CHK2* [60]. PARP inhibitor resistance in *BRCA1^−/−^* tumours develops through the restoration of HR via numerous mechanisms, including loss of 53BP1 and re-expression of wild-type *BRCA1* [61]. Importantly, while cancer-associated DDR gene mutations drive the development of the disease, they often lead to a dependency on a specific DNA repair pathway, knowledge of which can be exploited therapeutically in a synergistic or synthetic lethal approach.

### 3.1. Double-Strand Break Repair

**Figure 3:**
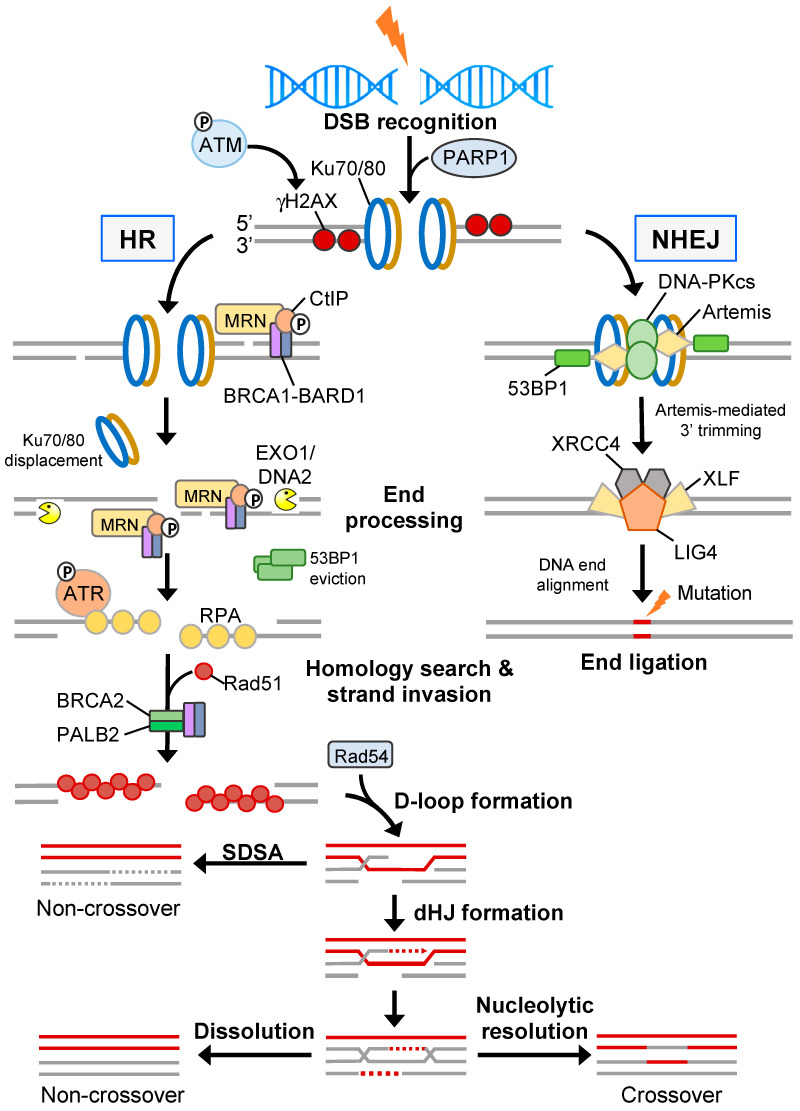
Homology- and non-homology-directed DSB repair. NHEJ is driven by the DNA-PKcs axis and re-joins two broken ends directly which usually leads to small DNA sequence deletions. Conversely, error-free HR involves extensive end resection generating 3′ single-stranded DNA tracts that are coated by RPA and subsequently displaced during Rad51-mediated strand invasion of a homologous DNA duplex. After D-loop extension, HR intermediates are mostly resolved through synthesis-dependent strand annealing (SDSA); however, in some cases double Holliday junction (dHJ) resolvases or dissolution is required resulting in crossover or non-crossover products, respectively.

Radiotherapy is cytotoxic via the induction of extensive two-ended DSBs. In normal cells, two-ended DSB repair is coordinated by two evolutionary conserved but mechanistically distinct pathways: canonical NHEJ (c-NHEJ) and HR (Figure 3) (reviewed in [62]). c-NHEJ occurs throughout all stages of the cell cycle, requires minimal processing of the DNA break ends, and is a rapid process but considered error-prone. In contrast, HR is error-free and requires extensive DNA end resection to generate sufficient ssDNA permitting invasion of the sister chromatid and can only occur during the late S/G_2_ phase of the cell cycle [63]. This exquisite cell cycle control of DNA repair pathway choice is principally regulated through the 53BP1-RIF1-shieldin pathway that acts in G_1_ phase to inhibit DNA end resection and BRCA1 recruitment to DSBs, thereby committing a cell to c-NHEJ activities [64]. During c-NHEJ, DSBs are sensed by the Ku70/Ku80 heterodimer which recruits the DNA-PKc/Artemis complex enabling DNA-PKc autophosphorylation, Artemis activation and endonuclease “trimming” of the non-compatible DNA ends [65]. DNA ligation is generally completed through the Ku-dependent recruitment of the XRCC4-LIG4 complex; however, in cases of increased DNA end complexity, other end-processing factors (e.g., PNKP and APTX) and DNA polymerases (e.g., POLμ and POLλ) are necessary for end re-ligation [66]. Since c-NHEJ is the most common DSB repair pathway, few cancers exhibit aberration in core c-NHEJ genes, presumably as the lack of c-NHEJ activity would be too detrimental. In line with this, elevated expression of DNA-PKc and the Ku heterodimers has been shown to promote radioresistance in several solid cancers [67]. In situations of a defective or inhibited c-NHEJ pathway, the cell may utilise the mutagenic back-up pathway alt-EJ (also called micro-mediated end-joining; MMEJ, or Theta-mediated end-joining; and TMEJ) to repair DSBs (reviewed in [68]). Here, the MRN complex, along with CtIP, generates short 3′ microhomology overhangs that enable DNA synthesis by Polymerase theta (Polθ) and DSB end ligation via the Ligase III/XRCC1 complex [69,70]. Polθ also suppresses HR by interacting with RAD51 thereby limiting RAD51-ssDNA nucleofilament formation and suppressing HR activity whilst stimulating alt-EJ [71]. In addition, the function of Polθ is critical for protecting against catastrophic genome instability brought about by excessive DNA end resection, as is the case in 53BP1-deficient cells [72].

During late S/G_2_ phase, when a sister chromatid is available as a homology template, DSB repair is preferentially mediated through the HR pathway. Here, the MRN (MRE11-RAD50-NBS1) complex removes Ku70/Ku80 and other DNA-bound protein components from break ends [73]. MRN also recruits and activates the sensor kinase ATM [74]. ATM phosphorylates numerous proteins, including H2AX on serine 139 (γH2AX) at sites of damaged chromatin, which in turn recruits MDC1 allowing signal amplification [75,76]. ATM also phosphorylates MDC1 enabling recruitment of the E3 ubiquitin ligase RNF8 which ubiquitylates linker histone H1 that is recognised by the UMI/MIU domains of RNF168. RNF168 ubiquitylates H2AK15, which in conjunction with H4K20me1/2, leads to the recruitment of the anti-resection factor 53BP1 [77,78,79,80,81,82]. Additionally, 53BP1 displacement is modulated by several mechanisms, including TIP60-mediated acetylation of H4K16 disrupting salt bridge formation between H4K20me2 and the Tudor domain of 53BP1 [83]; the recruitment of the BRCA1/BARD1 complex in post-replicative chromatin via the ARD domain of BARD1 preferentially interacting with H4K20me0 [84,85]; and SMARCAD1-dependent chromatin remodelling [86]. Initial short-range end resection by MRE11 within the MRN complex is followed by long-range end resection by two redundant pathways comprising EXO1 and BLM/DNA2 [87,88]. The resultant ssDNA is then rapidly coated by the heterotrimeric RPA complex leading to ATR-mediated checkpoint activation via CHK1 phosphorylation [89]. Through the action of the BRCA1/BRCA2/PALB2 complex [90], RPA is displaced and exchanged for RAD51 enabling nucleofilament formation, strand invasion and homology searching (Figure 3).

### 3.2. Interstrand Crosslink Repair by the Fanconi Anaemia Pathway

**Figure 4:**
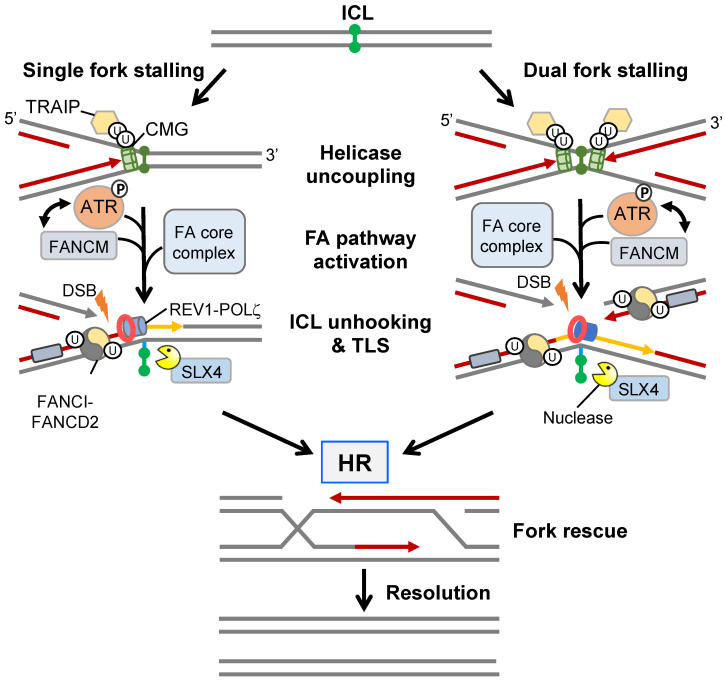
ICL repair by the FA pathway. The repair of an ICL in S-phase is initiated by a single fork stalling event or convergence of two stalled forks surrounding an ICL locus. After TRAIP-mediated disassembly of the CMG-replisome, the FANCM complex recognises the DNA crosslink and recruits members of the FA core complex and ATR, the latter facilitating a positive feedback loop with FANCM. The FA core complex monoubiquitylates dsDNA-bound FANCI-FANCD2 serving as the hub for the recruitment of structure-specific nucleases that, in cooperation with SLX4, incise the ICL site on both sides (5′ and 3′) of one parental strand generating a DSB in the opposite daughter chromatid. TLS polymerases (e.g., REV1 and POLζ) bypass the unhooked ICL restoring strand integrity of the first chromatid. The remnant ICL is subsequently removed by NER or BER, while the restored parental strand is used as a template for HR-mediated DSB repair.

Cisplatin is a common chemotherapeutic drug that induces the formation of cytotoxic interstrand crosslinks (ICLs). ICLs promote RS by preventing DNA strand separation, thereby blocking replication fork progression resulting in a one-ended DSB. The resolution of ICLs requires a complex interplay between the FA, TLS, and HR DNA repair pathways (Figure 4) and failure to resolve a single ICL can be cytotoxic to a eukaryotic cell (reviewed in [91]).

The sensing and stabilisation of an ICL-induced stalled replication fork is achieved through activation of the FA pathway. Initially, the translocase FANCM, together with associated FAAPs (FA associated proteins), prevent replication fork collapse and aberrant fork processing by activating ATR signalling leading to intra-S cell cycle arrest. FANCM also recruits the core FA complex which contains FANCL, an E3 ligase that monoubiquitylates FANCI and FANCD2 (collectively called the ID2 complex) [91]. Monoubiquitylated FANCD2 induces a conformational change that enhances binding of the ID2 complex to dsDNA effectively acting as a “clamp” whilst monoubiquitylated FANCI protects the ID2 complex from USP1-dependent deubiquitylation and degradation [92]. Monoubiquitylated ID2 forms filamentous structures extending kilobases in length that can be visualised in vivo as damage-associated foci and serves as a molecular platform that facilitates the recruitment of structure-specific nucleases and TLS polymerases [93]. For example, Ub-FANCD2 recruits SLX4 enabling the activation of various structure-specific nucleases including XPF-ERCC1, MUS81-EME1, and SLX1, all of which are known to contribute to ICL unhooking and the generation of a DSB on one chromatid [94,95]. The unhooked ICL, which is now attached to the DNA backbone via a short ssDNA tail, can undergo replicative bypasses via low-fidelity TLS polymerases REV1 and POLζ [96]. Following TLS polymerase disengagement, ID2-Ub is deubiquitylated by USP1-UAF1 [97], thereby allowing engagement of HR repair factors, such as RPA, RAD51, and GEN1 or SLX4-MUS81-EME1-SLX1 resolvases, that drive DSB repair.

In addition to ICLs, components of the FA/HR pathway promote replication fork restart, stabilisation and new origin firing in response to other replicative barriers such as R-loops [98] (DNA/RNA hybrids formed during transcription), nucleotide depletion [99], and DNA G4 quadruplexes [100] (reviewed in [101]). As such, tumours exhibiting FA/HR deficiencies respond favourably to non-ICL RS-inducing agents.

### 3.3. Single-Strand Break Repair Pathways

SSBs account for the most common type of DNA damage observed in cells and principally arise due to oxidation of deoxyribose (direct SSB) which is resolved via BER. However, they can also occur indirectly as a reaction intermediate during other DNA repair processes (e.g., NER, MMR, and ribonucleotide excision repair), as well as through the action of Topoisomerase I/II (reviewed in [102]). If not rapidly repaired SSBs can cause transcriptional pausing and RS, which can manifest into DSB formation through the instability and collapse of replication forks.

PARP1 is an ADP-ribosyl transferase required for sensing and orchestrating SSB repair (reviewed in [102]). PARP1 directly binds to the two ends of the SSB as a monomer, which allosterically relieves autoinhibition that promotes further PARP1 activation and auto-poly(ADP) ribosylation [103]. Poly(ADP) ribose (PAR) chains are both linear and branched, comprising of several hundred units that promote chromatin relaxation via the electrostatic repulsion of the DNA backbone and the recruitment of chromatin remodelling complexes (e.g., CHD4/NuRD and SMARCA5/SNF2H/ISW) [104,105]. A second crucial role for PAR chains is the recruitment of the XRCC1 complex which sequentially recruits most of the proteins required for direct SSB repair, including end-processing factors (POLβ, PNKP, TDP1, and APTX), gap filling (POLβ), and ligation (LIG3) enzymes [106]. Interestingly, PAR chains are rapidly degraded by PARG enzymes immediately after XRCC1 recruitment, whereby the XRCC1-BRCT2 domain is responsible for maintaining the complex at sites of SSBs [107]. Furthermore, PARP1 also recruits XRCC1 during BER-induced SSB repair which functions to limit PARP1 activity [108]. A failure to remove PARP1 results in reduced POLβ recruitment and ineffective BER [109]. As such, during BER, XRCC1 is an anti-PARP1 trapper functioning in an opposing manner to anticancer PARP inhibitors.

Whilst the most established role of PARP1 is in SSB repair, its functions extend to other DNA repair pathways (reviewed in [110]). Recently, PARP1 was shown to promote c-NHEJ through the recruitment of the Ku70/Ku80 heterodimer and retention of 53BP1-RIF1-shieldin to DSBs [111]. In addition, PARP1 may also be recruited to stalled replication forks where it poly(ADP-ribosyl)ates RECQ1 preventing premature fork restart [112]. Moreover, PARP1 recruits MRE11 and chromatin remodelling complexes allowing end-processing, replication restart, and HR-mediated repair [113,114,115]. Given the extensive DDR-related functions of PARP1, it is unsurprising that the development of PARP inhibitors has progressed at a rapid rate. However, similarities in the catalytic site between PARPs has meant that even the most clinically relevant compounds are not truly selective for PARP1/2. Interestingly, the effect of PARP inhibitors is greater than that of genetic deletion, principally because inhibition after DNA binding prevents auto-ribosylation that is required for PARP1 dissociation from DNA. Subsequently, PARP1 is effectively trapped leading to RS that requires multiple DDR pathways (HR, FA, and TLS) and DNA damage-dependent checkpoint activation for resolution [116]. This phenomenon is thus exploitable for targeted cancer therapies because tumours with loss-of-function mutations in HR genes are hypersensitive to PARP inhibitors. This has offered a therapeutic vulnerability that has been extensively explored clinically in *BRCA1/2* mutant patients but is now also being examined as a viable therapy in tumours with other specific genotypes, including those presenting with *ATM*, *PALB2*, *RAD51C*, and *RAD51D* mutations [117]. As mentioned previously, the major drawback of PARP inhibitors is the rapid onset of acquired resistance which is mediated through multiple mechanisms [118]. A better understanding of this will lead to novel therapeutic approaches that harness the power of drug targeting PARP enzymes, increasing the repertoire of potential therapeutic targets for CSCs.

## 4. DNA Damage Repair in Non-Transformed Stem Cells

As stem cells are long-lived and have the capability to self-renew and produce differentiated progeny, it is vital that they possess an efficient DDR to preserve the integrity of the genome. This is especially important for embryonic stem cells (ESCs), which can differentiate into any cell type including germline cells. For instance, human and mouse ESCs exhibited a lower mutational rate compared to their daughter cells suggesting improved genomic integrity processes [119]. One mechanism by which this is achieved in hESCs is via elevated expression of the cyclin-dependent kinase CDK1/2 and an increase in E2F1-mediated transcription of S phase genes [120]. Consequently, hESCs have a long S/G_2_ phase promoting repair of DNA lesions by the error-free HR pathway [120,121,122]. In addition, hESCs also have increased mRNA expression of DNA repair genes, including *BRCA1*, *WRN*, *BLM*, *FEN1*, *FANCL1*, and *FANCG,* inferring an augmented efficiency to repair damaged DNA [123]. Supporting this, hESCs can repair ionising radiation (IR)- and hydrogen peroxide-induced DNA damage at a more rapid rate compared to fibroblasts [123]. Furthermore, studies using induced pluripotent stem cells (iPSCs), which possess a short G_1_ phase, appear to exhibit increased dependency on the FA pathway as observed by a heightened expression of FANCD2 [124,125].

Most stem cells exhibit a reversible state of quiescence. Consequently, the reduced DNA replication rate and prolonged exit from the cell cycle limits genomic exposure to exogenous and endogenous DNA damage, thereby minimising the accumulation of DNA damage throughout the longevity of the cell. However, upon exiting quiescence stem cells proliferate rapidly adopting a state of hyper-transcription that is indicative of increased expression of chromatin remodelling and transcription machinery-related genes [126]. Hyper-transcription generates an increased number of transcription-replication conflicts that can present as sources of RS. For example, one such mechanism is through CHD1, a chromatin remodelling protein that is essential for transcription in mouse epiblasts [127]. CHD1 also interacts with ATM, PARP1, KAP1, and Topoisomerase 2β and remodels nucleosomes at GC-rich promoters to repair replication-induced DSBs that develop at transcription start sites [128]. By facilitating the resolution of DSBs, CHD1 can couple a global increase in transcriptional output to genome stability enabling sustained proliferation during embryogenesis. Furthermore, because of this rapid proliferation a substantial proportion of the mESC genome is not fully replicated before entry into mitosis. Surprisingly, genome stability is maintained, in part, through the action of the transcription factor MYBL2 that enables controlled replication initiation and ATM activation following RS [129]. Given that ATR, not ATM, is the principal sensor kinase to RS in somatic cells, this study suggests that the altered chromatin state of ESCs enables ATM to be activated in response to a wider range of DNA lesions.

Somatic adult stem cells are also thought to have an augmented DDR and principally engage c-NHEJ pathways. For example, mammary stem cells (MaSCs) isolated from whole body irradiated mice demonstrated an elevated resistance to DNA damage-induced apoptosis compared to luminal mammary epithelial cells [130]. This correlated with an increase in c-NHEJ activity and enhanced clearance of IR-induced γH2AX foci over time [130]. Likewise, mesenchymal stem cells repair IR-induced DNA lesions more rapidly than their differentiated counterparts through increased activation of ATM and DNA-PKc [131]. Analysis of murine hair follicle stem cells within the bulge niche after exposure to IR demonstrated a more efficient c-NHEJ response and resistance to DNA damage-induced apoptosis compared to differentiated basal epidermal cells [132]. Regarding the haematopoietic system, HSCs are mainly quiescent and predominantly utilise c-NHEJ for maintaining genome integrity and consequently exhibit increased rates of cancer-predisposing mutations [133,134]. This is supported by the fact that individuals with mutations in DNA repair proteins are severely immunocompromised and predisposed to cancer development [134,135]. In *FANCA*-deficient cells, exit of HSCs from quiescence in response to physiological stress factors, such as infection or blood loss, caused spontaneous DNA damage that could not be effectively repaired and eventually led to HSC depletion and bone marrow failure [136]. Moreover, HSCs derived from mice deleted in key c-NHEJ (*KU80, LIG4*), HR (*BRCA2, FANCD2*), and DNA damage sensing (*ATR, ATM*) genes showed defective reconstitution potential indicating impaired HSC self-renewal [137].

While the hypothesis that CSCs arise from the transformation of the equivalent normal somatic stem cell is an open question, comparisons of DNA repair competencies between the two cell types provides a crucial insight into the mechanisms responsible for intrinsic CSC drug resistance.

## 5. DNA Damage Repair in CSCs and Therapeutic Intervention

Similar to somatic stem cells, CSCs are thought to possess enhanced DNA repair capabilities including effective DDR pathway engagement, cell cycle checkpoint activation and a longer residence time in the quiescent G_0_ cell cycle phase. This ultimately leads to repair of chemotherapy-induced DSBs and chemoresistance. By this logic, targeting these pathways could re-sensitise CSCs to chemo/radiotherapy whilst simultaneously depleting the rapidly dividing bulk tumour cell population. Although numerous studies have demonstrated elevated DNA repair gene/protein expression in CSCs relative to the bulk tumour [138], there is still only a small number of investigations that mechanistically explore how the DDR is more efficient in CSCs (Table 2).

BCSCs isolated from triple-negative cell lines showed elevated expression of the MRN complex component RAD50, leading to enhanced HR activity [139]. Similarly, BCSCs derived from the ER^+^ MCF-7 cell line demonstrated reduced sensitivity to IR treatment which was correlated to increased HR activity as measured by the I-Sce/GFP reporter assay [140]. Interestingly, these BCSCs also showed a more effective RS response because hydroxyurea promoted replication fork degradation and DNA damage more extensively in the bulk MCF-7 population [140]. Supporting this, gene expression profiling of BCSCs from PDX breast models identified an enriched RS response gene signature compared to bulk tumour cells [141]. Since the ATR-CHK1 signalling pathway is critical for an effective RS response, these studies highlight the importance of its activation in BCSCs for genome stability. Furthermore, studies using a syngeneic model of basal breast cancer again demonstrated that, although both BCSCs and bulk cells were able to equally sense and respond to IR-induced DNA damage, BCSCs repaired the DNA damage at a more rapid rate [130]. In contrast, one study concluded that the DSB repair efficiency between BCSC and non-BCSC populations was similar [142]. However, in this case, analysis was conducted solely on mammospheres that comprise one BCSC and a mixture of progenitors rather than a BCSC-enriched population.

An enhanced DDR has also been observed in other CSCs. Lung CSCs showed an increased efficiency in resolving platinum-induced ICLs compared to the bulk cancer cells [143]. Moreover, these cells accumulated lower intracellular concentrations of cisplatin than differentiated progeny because of the reduced expression of the transporter genes *AQP2*, *ATP7B*, and *CTR1* [143]. Prostate CSCs are more chemoresistant triggering robust phosphorylation of CHK1 after etoposide treatment, which presumably correlates with an increased intra-S/G_2_ checkpoint response [144]. In radioresistant cervical cancer stem/progenitor cells isolated through spheroid-forming capabilities, several DDR genes with roles in different DNA repair pathways were upregulated, including a HR gene (*RAD51*)*,* a HR/single-strand annealing pathway gene (*RAD52*), c-NHEJ genes (*XRCC2* and *Ku70/80*), and genes associated with ROS metabolism (*CYBA2* and *SOD2*) [149]. However, this study did not demonstrate enhanced DDR kinetics nor characterise the enriched stem cell population. Finally, patient-derived GSCs displayed elevated expression of *CHK1*, *ATR,* and *PARP1* compared to the bulk cell population, and showed a more robust activation of the G_2_/M checkpoint after IR and increased radioresistance [145,146]. In addition, studies have also shown that GSCs exhibit a constitutively active CHK1-dependent DDR through increased endogenous RS, as well as preferentially repairing IR-induced DSBs by HR contributing to enhanced radioresistance [147,148]. Conversely, a study comparing the DNA repair kinetics in CD133^+^ GSCs and CD133^−^ non-GSCs showed similar levels of SSB and DSB repair and BER efficiencies [150]. However, the authors noted that the source of these GSCs were from glioma cell lines, which may exhibit phenotypic differences with patient-derived CD133^+^ GSCs.

Overall, the evidence supporting enhanced DNA repair in CSCs is limited and has generally relied on IR as the primary source of DNA damage, thereby linking augmented HR or NHEJ activities to drug resistance phenotypes. Much less is understood concerning RS-inducing chemotherapies that lead to replication fork protection, stalling, collapse, and one-ended DSBs. Despite this, advances have been made in identifying points of vulnerability within the DDR of CSCs that could be exploited for therapeutic intervention, particularly in the context of ATM, ATR, WEE1, and CHK1 targeting [151].

### 5.1. Drug Targeting the ATR-CHK1-WEE1 Checkpoint Pathway in CSCs

Signalling between the DNA damage sensor kinases ATM/ATR and the cell cycle regulator proteins CHK2/CHK1 is critical for cell cycle arrest following DNA damage, allowing time for DNA repair. The ATM-CHK2 axis is principally activated after DSBs. ATM phosphorylates CHK2 on Thr68 leading to dimerisation, autophosphorylation, and inactivation of the phosphatases Cdc25A/C. Consequently, the lack of Cdc25A/C-mediated dephosphorylation of CDK1/2 promotes G_1_/S and G_2_/M checkpoint activation. Conversely, the ATR-CHK1 pathway is triggered by ssDNA generated in response to numerous types of RS, including transcription-replication collisions, nucleotide depletion, topoisomerase poisons, and ICL-inducing agents, and activates the intra-S and G_2_/M checkpoints. Specifically, ATR phosphorylates CHK1 at Ser345, resulting in the inactivation of Cdc25A/C. Additionally, ATR phosphorylates the WEE1 kinase, which triggers an alternative pathway of CDK1/2 inhibition. Importantly, as the ATR-CHK1 pathway is also critical for replication fork protection and stability, targeting the ATR-CHK1 pathway could lead to multiple points of vulnerability that synergise with therapeutic agents which induce RS. Since CSCs have been shown to display enhanced DNA damage-dependent checkpoint activation, this concept has gained significant traction as a novel CSC-directed therapeutic approach.

Earlier studies indicated that targeting the CHK1/CHK2 axis with a non-selective dual inhibitor was able to sensitise CD133^+^ glioma CSCs to radiotherapy and reduce their in vivo tumorigenicity [145,146,152]. Subsequently, more selective ATR and CHK1 inhibitors demonstrated in vitro potency by acting as a radiosensitiser in patient-derived glioma CSCs [146]. Similarly, ESA^+^CD24^+^CD44^+^ pancreatic CSCs and NSCLC sphere cultures exhibited augmented ATR-mediated CHK1 phosphorylation compared to non-stem cell counterparts and combination therapy of CHK1 inhibition with gemcitabine or cisplatin increased DNA damage conferring CSC chemosensitivity [153,154]. The mechanism of action of CHK1 inhibition appears to be related to the abrogation of the DNA damage-induced G_2_ checkpoint, reduced RAD51 recruitment and defective HR [155]. Although effective, the clinical development of these checkpoint kinase inhibitors has been hampered by disappointing drug efficacy in cancer patients as well as off-target patient cardiotoxicity issues [156,157]. An alternative approach is drug targeting WEE1. Inhibition of WEE1 was shown to sensitise trastuzumab-resistant BCSCs to chemotherapy-induced apoptosis, and co-administration of an ATR and WEE1 inhibitor showed an elevated synergistic cytotoxic effect in BCSCs isolated from an orthotopic breast cancer xenograft mouse model [158,159]. Mechanistically, this was due to a lack of intra-S and G_2_/M checkpoint, thereby allowing cells with under-replicated and damaged DNA to progress prematurely through the cell cycle leading to mitotic catastrophe. Moreover, since CHK1 signalling is controlled by ATR activation, co-administration of RS agents should potentiate cytotoxicity. This is particularly relevant for CSCs where increased levels of endogenous RS may be an inherent characteristic [160]. Indeed, a recent study used an inhibitor screen on colorectal CSCs from 27 patients and identified the CHK1 inhibitor, LY2606368, as a potent in vitro and in vivo agent [161]. LY2606368-sensitive colorectal CSCs exhibited elevated amounts of RS and endogenous DNA damage due to defective ATR-mediated checkpoint activation and replication-induced catastrophe. Interestingly, since wild-type *p53* status appears to protect against the effects of LY2606368, *p53* mutational status, along with phosphorylation of RPA and ATR, could act as in vivo biomarkers for predicting patient treatment responses [161]. Another highly selective CHK1 inhibitor, rabusertib/LY2603618, also reversed ATR-dependent chemoresistance in colon sphere cultures to genotoxic agents that primarily induce RS as their mode of action [162]. Following the principle of synthetic lethality, ATR/CHK1/WEE1 inhibitors can be further exploited in tumours that exhibit either *ATM* (~5%) or *p53* (~50%) mutations. These cancer cells cannot activate CHK2-mediated G_1_/S checkpoint and are consequently dependent on the S and G_2_/M checkpoint for DNA repair [163]. Therefore, this provides a vulnerability that can be exploited with ATR/WEE1 inhibitors.

### 5.2. ATM Inhibitors in CSCs

ATM has also been proposed as a valid drug target against CSCs, especially in the context of radiotherapy. However, like ATR, there are currently only a small number of ATM inhibitors that have been tested in CSCs. Nevertheless, a study investigated the in vivo propagation of patient glioblastomas via orthotopic implantation of GSCs into NOD-SCID mice and demonstrated that ATM inhibitors were able to radiosensitise both adult and paediatric tumours [164]. These findings are particularly significant as the clinical ATM inhibitor, AZD1390, penetrates the blood-brain barrier (BBB) improving the survival of in vivo glioblastoma tumour models [165].

### 5.3. PARP Inhibitors in CSCs

Overexpression of PARP1 has been observed in colorectal CSCs compared to matched normal stem cells, and although PARP inhibitor monotherapy failed to kill colorectal CSCs, combination treatment with either 5-FU, oxaliplatin, or leucovorin promoted apoptosis [166,167]. Combining PARP and ATR inhibitors has also proven successful for enhancing in vivo efficacy in preclinical models of PARPi-resistant and *BRCA1/2*- and *ATM*-deficient ovarian and colon CSC models, presumably because PARP inhibition creates ssDNA replication gaps and damage in S-phase, which ultimately requires checkpoint and HR pathway engagement for repair [116,168,169,170]. Whilst ATR/PARP inhibitors have raised some concerns regarding haematological toxicities, sequential dosing regimens could provide a clinical approach to minimise these side effects [171]. Moreover, current research using CRISPR chemogenomic screening has identified additional synthetic lethal interactions, including RNaseH2, that synergise with ATR/PARP inhibition, thereby providing novel approaches [172]. It will be interesting to see if the findings from bulk tumour cells are reproducible within the chemoresistant CSC population.

Whilst PARP activity and the formation of PAR chains is central in orchestrating numerous DNA repair pathways, the activity of PARP itself appears to be differentially regulated in cancer cells. The catalytic activity of PARP requires the consumption of nicotinamide adenine dinucleotide (NAD^+^) as a substrate. Nicotinamide (NAM) functions as both the precursor and by-product to NAD^+^-consuming reactions; however, elevated concentrations of NAM can act as an endogenous PARP inhibitor. This has consequences for DNA repair because exogenous NAM sensitised breast cancer cells to cisplatin and IR [173]. Indeed, it has been shown that administration of NAM prior to radiotherapy improves disease-free patient survival rates in some cancer types such as bladder and prostate [174]. One mechanism by which this is thought to occur is by improving blood flow in hypoxic tumours, thereby increasing oxygenation and presumably the number of cytotoxic DSBs induced by IR [175]. It would thus be interesting to determine if NAM also promotes chemo/radiosensitivity through additional mechanisms related to its role in PARP inhibition, thereby possibly indicating that NAM is directly involved in modulating the DDR.

Since NAM has multiple effects on the cell, it is critical that cellular levels are controlled. To reduce excess amounts, NAM is methylated by nicotinamide N-methyltransferase (NNMT), effectively removing it from the NAD^+^ biosynthesis salvage pathway [176]. Interestingly, overexpression of NNMT has been observed in multiple tumour types as well as in CSCs derived from glioma, bladder, colorectal, osteosarcoma, and lung cell lines [176,177,178]. Although the mechanisms have not been elucidated, elevated NNMT is thought to promote the CSC phenotype by reducing DNA methylation and inducing expression of EMT-associated genes [179]. It is therefore plausible that NNMT, through regulation of NAM levels, may also contribute to chemoresistance by promoting the effectiveness of PARP-dependent DNA repair mechanisms in CSCs. Hence, NNMT inhibitors [180]—alone or in combination with olaparib—could provide interesting new avenues for CSC therapies, particularly in the efforts to prevent acquired PARPi resistance.

### 5.4. Drug Targeting HR Factors in CSCs

Targeting specific downstream HR effectors has shown promise in chemosensitising CSCs. For example, increased *RAD51* mRNA expression is frequently observed in multiple types of CSCs compared to differentiated cancer cells, and inhibition with resveratrol or siRNA knockdown reduces CSC viability [141,181,182,183,184]. Indeed, while BCSCs derived from *BRCA1*-mutant TNBC cell lines (SUM149) were resistant to olaparib, knockdown of *RAD51* enabled re-sensitisation [181]. Because of this, drug targeting RAD51 has become a major line of investigation as an alternative approach in disrupting HR independent of the sensor/checkpoint DDR kinases. Small molecule tool compounds that inhibit RAD51 nucleofilament formation, or prevent the dissociation of RAD51 from ssDNA, are in preclinical development [185]. One compound, RS-1, is especially toxic in cancer cells that express high levels of RAD51 but low levels of RAD54L, a protein that removes RAD51 from dsDNA. RS-1 exhibited high tolerability in mice, possessed anticancer activity in mouse prostate cancer xenograft models and was more potent in low expressing RAD54L cells [186]. Accordingly, high levels of RAD54L confer resistance to RS-1. Additionally, using a structure-led fragment-based approach, CAM833 was recently identified as a small molecule that prevented RAD51-BRCA2 interactions and hence nucleofilament formation, and synergised with IR and PARP inhibitors in cancer cells [187]. This approach, however, is presumably restricted to *BRCA2* wild-type cancer cells. Regarding CSCs, one study has shown that combining RAD51 inhibition and genetic suppression of the PARP1 coactivator SAM68 increased the susceptibility of the BCSC-rich mammosphere cultures to RS, thereby reducing their cell viability [184]. Overall, these studies exemplify the crucial role of RAD51 recombinase in mediating chemoresistance and maintaining HR activity in CSCs, even in circumstances of BRCA1/2 deficiency.

### 5.5. Drug Targeting c-NHEJ Factors in CSCs

Although the S and G_2_/M checkpoint and HR repair pathways appear to be the most prominent mediators of CSC chemoresistance, the error-prone c-NHEJ pathway may also function as an additional therapeutic barrier. DNA-PK is a key c-NHEJ protein, with preclinical DNA-PK inhibitors demonstrating potent antitumoral activity by increasing the sensitivity of various cancer cell lines to DSBs induced by chemo/radiotherapy [188,189,190]. Although studies are few in CSCs, treatment of cell line-derived BCSCs with a highly selective DNA-PK inhibitor (NU7441) has demonstrated reduced DNA repair leading to chemo/radiosensitivity [191]. In GSCs, the catalytic subunit of DNA-PK—DNA-PKcs—is more highly expressed than ATM, implying a crucial role in genome stability [192]. Moreover, the expression of DNA-PKcs is elevated in patient-derived GSCs compared to matched non-CSCs, and treatment with NU7441 reduced in vitro tumour stemness and tumour initiation sensitising GBM xenografts to radiotherapy [193]. Although it is currently unclear whether NU7441 can penetrate the BBB, the development of the melanotransferrin antibody and tamoxifen-conjugated nanoparticles have enhanced delivery of other chemotherapies in in vitro BBB models [194], at least providing proof of principle that drug targeting DNA-PK has clinical utility.

### 5.6. DNA Repair Gene Splicing as a Mechanism of CSC Chemoresistance

Most therapeutic approaches are focused on directly targeting enzymes involved in sensing and repairing DNA lesions, however strategies that lead to indirect regulation of key DNA repair proteins are also a viable option. Modulating mRNA splicing is one such approach because mRNA splicing fidelity and appropriate transcript isoform expression is deregulated in cancer. Targeting the splicing machinery has thus become increasingly researched in cancer therapeutics because RNA-binding proteins (RBPs) involved in splice site recognition and spliceosome activation are mutated or upregulated in multiple cancer types [195,196,197]. This includes members of the serine/arginine (SR)-rich splicing factor proteins and heterogeneous nuclear ribonucleoproteins (hnRNPs) [198,199].

Surprisingly, the role of splicing within CSCs has received very little attention relative to bulk cancer cells [195]. However, examination of the transcriptome of human AML progenitors identified a distinct splicing signature that could distinguish between normal and malignant progenitors, suggesting that deregulated splicing in CSC-like cells contributes to disease progression in AML [200]. Supporting this, treatment of xenograft models of human AML with 17S-FD-895, a small molecule compound that modulates splicing, reduced leukemic stem cell numbers and self-renewal potential while the survival of normal HSCs was unaffected [200]. This suggests that therapeutic targeting of splicing could be a novel approach for CSCs. Indeed, MDS patients frequently exhibit mutations in the spliceosome machinery that is thought to be pathogenic, with conditional expression of *Srsf2-P95H* within murine HSCs sufficient to drive MDS development [201,202]. While the identities of the RBPs that contribute to this switch in splicing during AML progression are yet to be determined, other RBPs have since been implicated in CSC biology. For example, both SRSF3 and TRA2β are frequently upregulated in breast cancer and regulate the splicing of *CD44* leading to the expression of the CD44v isoform that is associated with increased stemness and metastatic traits [203,204,205]. Likewise, SRSF1 promotes EMT and cell motility in breast and pancreatic cancer [206,207] and, in APC-deficient colorectal cancer murine models, SRSF1 expression correlates with CSC marker expression maintaining a stemness phenotype by promoting cellular plasticity [17]. Taken together, this suggests that elevated levels of RBPs in cancers alter splicing promoting stem cell-like traits, and as such, could contribute to the generation and maintenance of the CSC population.

Interestingly, there has been a growing appreciation for the role of RBPs and splicing modulation in maintaining genome integrity. The activity of RBPs is controlled by extensive post-translational modifications, which is especially significant following DNA damage. Quantitative proteomic analyses of ATM/ATR substrates after IR identified RNA processing as a significantly enriched Gene Ontology category, thereby linking for the first time RNA metabolism to the DDR [208]. Supporting this, SRSF1 is phosphorylated after UV radiation, aphidicolin, and etoposide treatment in a partially ATM/ATR-dependent manner [209], and depletion of SRSF1 or inhibition of the SF3B complex with pladienolide B promoted R-loop formation, a type of RS that if unresolved can lead to DSB formation [210,211]. Furthermore, other studies have highlighted the role of RBPs in the DDR. For example, the use of a global siRNA screen identified the RBP, RBMX, as a novel HR factor that promoted resistance to DNA damaging agents [212]. Intriguingly, although RBMX was recruited to sites of DSBs, the RRM (RNA recognition motif) domain was dispensable for this. RBMX was also shown to promote *BRCA2* expression; however, whether this was due to splicing was not examined [212]. Likewise, SF3B1 supports HR-mediated DSB repair, with the most common *SF3B1* cancer-associated mutation K700E promoting genome instability and DNA damage [213]. SRSF2-P95H, another common cancer-associated mutation, was shown to promote DNA damage and ATR-CHK1 pathway activation through aberrant R-loop formation [214]. Consequently, SRSF2 mutant leukemic cells exhibited increased synergistic sensitivity to the CHK1 inhibitor UCN-01 and spliceosome inhibitor Sudemycin D6 [215]. A similar synergy was also observed in pancreatic ductal adenocarcinoma CSCs treated with gemcitabine and a combination of splicing inhibitors [216]. While the mechanism behind this synergy was not elucidated, it suggests that these cells could not efficiently resolve RS induced by gemcitabine.

Other studies have linked splicing/RBPs to the DDR response and CSC biology. The expression of the RBPs TRA2α/TRA2β correlated with invasiveness in breast cancers and a poor prognosis in cervical cancer [205]. Dual *TRA2α/TRA2β* knockdown resulted in reduced expression of full-length *CHK1* and accumulation of DNA damage [217]. Likewise, transcriptomic analysis of cervical cancers showed increased expression in tumour cells compared to matched normal tissues of the RBP *SRSF6,* and that this corresponded with an increase in alternative splicing of DDR genes [218]. Intriguingly, SRSF1 binds to and stabilises *RECQL4* mRNA which is important because RECQL4 is a master regulator of genome stability through regulating DNA replication and multiple DNA repair pathways, including c-NHEJ, HR, BER and NER [219,220]. RECQL4 expression was elevated in GSCs compared to bulk tumour cells, which was functionally relevant because knockdown reduced GSC proliferation and induced spontaneous DNA damage [221]. This suggests that high levels of RECQL4 in GSCs contributes to genome stability because SRSF1 stabilises *RECQL4* mRNA.

Given the critical role of splicing in cancer, numerous approaches have been suggested for therapeutic intervention. Inhibition of the core spliceosome with compounds such as FR901464 and pladienolide B are suitable reagents for in vitro analysis, however, concerns of narrow therapeutic windows and toxicities have limited clinical application. Similarly, drugging specific splicing factors and RBPs is challenging because they lack a catalytic domain. An alternative approach is to target the activity of enzymes that post-translationally modify components of the splicing machinery. One such example is PRMT5, an arginine methyltransferase that is required for normal stem cell and CSC function [222,223,224,225]. Specifically, PRMT5 symmetrically dimethylates arginine residues predominantly at RG or RGG residues, motifs that are highly enriched in RBPs and intrinsically disordered regions [226,227]. Indeed, the core spliceosome components, SmB and SmD1/3, are key PRMT5 substrates enabling the assembly of the mature core small nuclear ribonucleoproteins (snRNPs) [228]. The importance of PRMT5 for splicing has since been demonstrated by numerous approaches including genetic knockout and enzymatic inhibition [223,224,225,229,230]. More importantly, there is a growing appreciation that PRMT5 regulates splicing events that contribute to genome stability in stem cells. For instance, using selective deletion of *Prmt5* in neuronal stem/progenitor cells via the *Nestin*-Cre transgene, Guccione and colleagues identified *MDM4*, a negative regulator of the p53 response, as a PRMT5-dependent alternative splicing event [225]. *Prmt5* deletion resulted in the skipping of a weak 5′ splice acceptor site within the *MDM4* pre-mRNA transcript leading to exon 7 exclusion and the generation of *MDM4S,* a less stable isoform than the canonical full-length *MDM4* which resulted in reduced levels of functional MDM4 protein and p53-induced apoptosis [225]. Subsequently, it was shown that deletion of *Prmt5* in fetal and adult haematopoietic progenitors resulted in widespread disruption of splicing events enriched for intron retention and exon skipping. Significantly, several chromatin modifiers that regulate HR repair were aberrantly spliced, including *TIP60*, leading to the generation of the shorter *TIP60β* isoform that exhibited reduced acetyltransferase activity [223]. Given that *Prmt5* knockout in HSCs/progenitors showed defective HR repair and accumulated DNA damage, these findings highlight aberrant splicing as a major contributing factor to genome instability that is driven by deficient or abnormal PRMT5 activity. Interestingly, PRMT5 also directly regulates the activity of TIP60 through the dimethylation of RUVBL1 [231], implying that the role of PRMT5 in HR repair in cancer cells is multifactorial. In addition to normal adult stem cells, PRMT5 also regulates splicing within CSCs. For instance, inhibition of PRMT5 in GSCs by two orthogonal-acting inhibitors, GSK591 and LLY-283, caused splicing deregulation and reduced GSC viability. Moreover, this study was also able to define a splicing signature in GSC lines that was predictive of a PRMT5 inhibitor response. As PRMT5 inhibitors are in clinical trials for various cancers [232], studies that identify putative patient populations will be critical to ensure therapeutic success without associated toxicities. In line with this, MDS and AML cells with SRSF2-P95H mutations display a synthetic lethal interaction with PRMT5 inhibitors [233].

To summarise, cancers require both a tight level of splicing fidelity in essential genes and the induction of alternative splicing events to drive and maintain a malignant phenotype. Supporting this, key splicing proteins and enzymes that control splicing events are upregulated in many cancers. Although research into the role of splicing in CSCs is in its infancy, the DDR appears to be particularly relevant and suggests that maintaining the fidelity of DNA repair gene splicing and pathway engagement in CSCs could be an important mechanism of drug resistance. Since antisense oligonucleotides (ASOs) that mimic the *MDM4* splicing event induced by impaired PRMT5 activity impairs tumour growth in vivo [234], combination approaches involving low-dose chemotherapies and small molecule-induced splicing deficiencies could be an effective way of eliminating CSCs.

## 6. Challenges and Opportunities for the Future

Clearly, eradicating CSCs is critical for improving the efficacy of anticancer regimens. The presence of robust DDR pathways and checkpoint activation capabilities endows CSCs with a survival advantage over non-CSCs, while simultaneously conferring a unique therapeutic vulnerability. Combining new therapeutic strategies, such as targeting DDR kinases and checkpoint regulators, in combination with DNA damage therapy could overcome the cytoprotective function of the DDR, consequently re-sensitising tumours to chemo/radiotherapy. Despite CSCs being increasingly susceptible to DDR signalling abrogation, there is still only a limited number of studies that have investigated the effects of small molecule DDR-related agents, either alone or in combination with other inhibitors/DNA damaging agents, in this cell type. Moreover, the DDR is incredibly complex operating within multiple positive and negative feedback loops all of which are influenced by numerous factors. This includes the extent of open/closed chromatin influencing the level of DNA damage and repair mechanisms, enzymatic activities other than phosphorylation, and the requirement of alternative splicing or splicing fidelity for elevated DDR transcript expression that promotes chemoresistance. It is hoped that further progress in these areas will offer new therapeutic approaches that, if successful in preclinical mouse models, could become a viable option for treating the patient population.

One way in which these areas of research could be addressed is through identifying novel synthetic lethal relationships with specific DDR small molecule inhibitors and existing chemotherapeutic agents via CRISPR-Cas9-mediated gene knockout screens. This has been exemplified by the Durocher laboratory, which performed 31 CRISPR-Cas9 viability screens against 27 genotoxic agents in the non-transformed RPE-1 cell line [235]. Using this systematic approach, they identified several new mechanisms by which specific agents engage a particular DNA repair pathway leading to either chemotherapy sensitivity or resistance. For example, the cytotoxic mechanism of the G4-quadruplex stabilising agent pyridostatin unexpectedly involved the poisoning of TOP2, and thus functions in a similar manner to that of etoposide, thereby offering alternative approaches in the case of etoposide resistance. Likewise, ERCC6L2, a gene mutated in MDS and leukaemia that is associated with bone marrow failure, was identified as a new NHEJ factor recruited to chromatin after IR promoting DNA end-joining independent of the 53BP1-RIF1-shieldin pathway [235]. These genome-wide approaches are therefore highly effective in characterising DNA repair mechanisms and novel DNA repair proteins. These can then be exploited through the identification of therapeutic vulnerabilities, either by combinatorial approaches or establishing genetic backgrounds in a cancer type that will be more susceptible to a specific DNA damaging agent.

Although the same approach in CSCs will no doubt provide critical new insight into targetable vulnerabilities, such studies are relatively unexplored because CRISPR screens using DNA damaging agents require prolonged cell culture conditions for phenotypic evaluation. This is particularly challenging in CSC models because, by their nature, they are subject to spontaneous differentiation producing progenitors. Indeed, the mammosphere assay that is used routinely for in vitro measurement of BCSC self-renewal and proliferation requires just 5 days for extensive progenitor/bulk cell expansion. Consequently, determining CSC-specific synthetic lethal interactions is confounded by the heterogeneity of the cell population in standard CSC culture conditions. Moreover, robust statistical analysis on sgRNA sequence abundance generally requires an extensive number of cells, which can be practically problematic for minor CSC subpopulations. Indeed, few tumour types offer large-scale CSC enrichment procedures, with glioma an exception since GSC lines can grow in culture as adherent stable cultures yet still retain stemness properties [236,237]. Even so, such cell lines have yet to undergo a CRISPR-based genomic screen with DNA damaging chemotherapies or a sgRNA library directed towards DDR proteins. In the future, with continuing advancements in multiplexed CRISPR/Cas9-mediated genome editing, it might be feasible to conduct CSC-directed CRISPR screens in vivo. For example, combining the integrated expression of sgRNAs downstream of a CSC-specific gene promoter with Tuba-seq barcode labelling could enable discrimination between CSCs and bulk tumours cells during sequencing-based counting of sgRNAs, thereby identifying CSC-specific synthetic lethal events [238].

One major consideration required for effective eradication of CSCs is the concept of CSC plasticity which implies that therapeutic interventions must target both the rapidly proliferating bulk tumour cells and the CSC compartment. Indeed, to prevent chemotherapy-induced expansion of the CSC population, combination approaches that compromise multiple DNA repair pathways and/or target a specific epigenetic/genetic vulnerability will need to be considered. Likewise, inter- and intratumoral CSC heterogeneity necessitates that a repertoire of anti-CSC DDR targets will most likely be required for complete CSC eradication. Addressing this presents a major challenge for researchers in the CSC field, particularly as cell surface markers that delineate CSCs have their limitations. scRNA-Seq may identify clonal CSC populations with deregulated DDR pathways, but will only be able to consider genetic aberrations that lead to transcriptional changes and are thus limited in clinical application. Furthermore, while PARPi resistance is widely attributed to the lack of clinical efficacy in bulk tumour cells, it is apparent that CSCs can also develop similar resistance mechanisms. Regarding next-generation DDR kinase inhibitors, treatment of HeLa and MDA-MB-231 bulk cells with the WEE1 inhibitor AZD1775 led to resistance through upregulation of Myt1, a CDK1 kinase that is functionally redundant with WEE1 [239]. As Myt1 appears to promote differentiation, at least in non-transformed neuronal and intestinal stem cells, drug targeting could incidentally promote CSC survival [240,241]. Likewise, ATR inhibition resistance mechanisms in mESCs involved the loss of Cyclin C or CDK8, components of the RNA polymerase II mediator complex, resulting in reduced transcription-associated RS [242]. To date, it is unclear if any of these DDR kinase inhibitor resistance mechanisms are also operating within the CSC population. Finally, alternative approaches to target the DDR may emerge as the interplay between DNA repair and cancer cell survival is further unravelled. For instance, an exciting development yet to be examined in CSCs, is the role of the back-up DNA repair pathway alt-EJ. Although alt-EJ is considered a relatively minor DNA repair pathway in NHEJ/HR-proficient cells, it is now gaining significant traction as a druggable pathway in HR-deficient cancers. For example, deletion of Polθ is synthetic lethal with various DNA repair genes, including *BRCA1/2* mutations [68,71,243] and can synergise with PARP inhibitors in HR-defective cancers [71]. Furthermore, Polθ inhibitors have also been shown to re-sensitise chemoresistant and radioresistant lung cancer cells, as well as sensitising cancer cells that have acquired PARPi resistance [244,245,246]. Since Polθ is overexpressed in many cancer types and is correlated with a poor patient prognosis—and that drug targeting might be cancer-specific given that normal tissues are HR-proficient [68]—small molecule compounds have progressed at a rapid pace. With the Artios compound (ART4215) initiating patient recruitment for a phase I trial in January 2023 (clinical trial identifier: NCT04991480), preclinical studies into the role of Polθ in CSCs could lead to exciting new treatment strategies.

The splicing landscape is an especially understudied area of CSC biology even though small molecule inhibitors against key enzymes that regulate splicing, such as PRMT5, are in clinical trials for cancer treatment. While analysis of differential splicing events in CSCs is easily achievable, a deeper mechanistic understanding is more challenging due to current methodological limitations. Indeed, even though iCLIP is a powerful approach that enables an unbiased identification of mRNA transcripts binding to a specific RBP, tens of millions of cells are required for robust data collection. As other genomic approaches such as sc-RNA-Seq and low cell number ChIP via the Cut and Tag/Run methodology are now becoming routine, it would be exciting to see similar developments in the RBP space that would make iCLIP in CSCs more feasible. With the improved delivery of ASOs, drug targeting specific splicing events in bulk tumour cells and CSCs leading to effective clinical responses could become a reality, as it has for some neurological diseases [247].

Most studies investigating DNA repair in CSCs have been conducted using in vitro cell line models or xenograft transplantation in immunocompromised mice. Hence, another important question is how does targeting the DDR and/or its regulatory components influence the CSC niche? This is particularly interesting regarding DNA repair because preclinical compounds that deregulate splicing induce the formation of neo-epitope production that are immunogenic [248]. Furthermore, olaparib-induced activation of the cGAS-STING pathway evokes an antitumoral immune response in bulk tumour cells that is a critical component for an effective treatment response [249,250]. As such, combining DNA damaging chemotherapies with splicing inhibitors could provide a more immune-modulatory effect within the stem cell niche. The continued development of CSC models that consider the CSC tumour microenvironment, such as humanised PDX murine models and advanced organoid culture systems [251,252,253], will also reveal exciting new discoveries within the DNA repair space.

Finally, is there a way to target CSCs through modulation of DNA repair efficiencies without adversely affecting normal somatic stem cells? This is particularly relevant for HSCs, which are highly dependent on maintaining genome stability for survival. One recent advancement that may enable tumour selectivity is nanoparticle technology [254]. These novel platforms involve encapsulating drugs in polymer-lipid hybrid nanoparticles, which recognise antigens associated with CSCs, such as CD44, ALDH1, and CD133, and have been shown to target BCSCs and colorectal CSCs [255,256]. If CSC heterogeneity can be overcome through the synthesis of nanoparticles expressing a range of ligands, then this approach could have far-reaching potential for cancer therapies. However, utilising nanomedicines for targeting CSCs is, at present, restricted by the deficiency of a universal CSC biomarker essentially making it difficult to gauge the efficacy of CSC-directed therapies.

## 7. Conclusions

To conclude, there is a growing body of evidence that identify CSCs as the culprit of tumour drug resistance and recurrence. Critically, CSCs appear to show increased DNA repair competency compared to differentiated bulk cancer cells which presents viable CSC-targeted therapeutic opportunities within the DDR. The focus of future trials should therefore capitalise on these CSC-specific DDR dependencies and include combinatorial approaches with chemo/radiotherapy to improve cancer patient outcome.

## Figures and Tables

**Table 1:** Methods for the isolation or enrichment of human and murine breast cancer stem cells using cell surface and intracellular markers.

Strategy	Enriched Cells (%)	Reference
EpCAM^+^CD24^lo/−^CD44^+^Lin^−^	0.60	[3]
CD24^lo/−^CD44^+^	1.32–5.00	[22]
CD133^+^	2.00–5.90	[22]
EpCAM^+^CD49f^+^	Unknown	[21]
CD29^+^CD24^hi^Lin^−^ (murine)	5.00–10.00	[20]
ALDH1^+^	3.00–10.00	[24]
CD24^+/low^Sca-1^−^CD49f^hi^ (murine)	20.00–30.00	[13]
ABCG2^hi^	0.2–5.00	[23]

**Table 2:** Enhanced DNA repair efficiency in connection to CSC chemo/radioresistance mechanisms.

DDR Pathway	Mechanism	DNA Damaging Agent(s)	CSCs	References
HR	Increased RAD50 (MRN) expression	Mitomycin	BCSCs (tumoursphere)	[139]
HR RS response	ATR-CHK1 axis	IR, hydroxyurea	BCSCs (ALDH1^+^)	[140]
HR RS response	BMI1-RAD51 axis	Cisplatin	BCSCs (ALDH1^+^)	[141]
HR ICL repair MMR	Reduced intracellular [cisplatin]; increased gene expression (*PMS2*, *ERCC1*, *MLH1*, *MSH2*)	Cisplatin	Lung CSCs (chemoresistant cell line)	[143]
DSB repair	Enhanced CHK1 activation	Etoposide, docetaxel	Prostate CSCs (tumoursphere)	[144]
SSB repair DSB repair	Enhanced CHK1/2 activation	IR	GSCs (CD133^+^)	[145,146]
RS response	ATR-CHK1 axis; constitutive RS	IR	GSCs (CD133^+^)	[147]
HR	SPT6-mediated HR	IR	GSCs (CD133^+^)	[148]
